# Development and characterization of 16 novel microsatellite markers by Transcriptome sequencing for *Angelica dahurica* and test for cross-species amplification

**DOI:** 10.1186/s12870-020-02374-8

**Published:** 2020-04-08

**Authors:** Qianqian Liu, Zuyu Lu, Wei He, Fang Li, Wenna Chen, Chan Li, Zhi Chao, Enwei Tian

**Affiliations:** 1 Affiliated Cancer Hospital & Institute of Guangzhou Medical University, Guangzhou, 510095 China; 2 School of Traditional Chinese Medicine, Southern Medical University, Guangzhou, 515005 China; 3 College of Landscape and Ecological Engineering, Hebei University of Engineering, Handan, 056038 China

**Keywords:** SSR markers, RNA-Seq, *Angelica dahurica*, Population genetics, Genetic diversity

## Abstract

**Background:**

*Angelica dahurica* (Apiaceae) is an important herb in traditional Chinese medicine. Because of its important medicinal and economic values, its wild resources were over-exploited and increasingly reduced. Meanwhile, the diversity of cultivars of *A. dahurica* has decreased as a result of long-term artificial cultivation. However, there are no population genetics studies of natural *A. dahurica* reported yet, especially for using microsatellite markers (SSRs) to investigate population genetics of the species.

**Results:**

Sixteen polymorphic EST-SSR loci were isolated from *A. dahurica* with transcriptome sequencing technology (RNA-Seq). The number of alleles varied from 2 to 15 per polymorphic locus over populations with the observed and expected heterozygosities ranging from 0.000 to 1.000 and from 0.000 to 0.829, respectively. Significant deviations from Hardy–Weinberg equilibrium were observed at 8 loci. Tests of linkage disequilibrium showed 11 informative locus pairs were significant across all populations. Cross-species amplification showed that 14 out of 16 SSR loci have the transferability in cultivar-*A. dahurica* cv. ‘Hangbaizhi’ and *A. decursiva*.

**Conclusions:**

The 16 newly developed loci microsatellite primers with RNA-Seq will be useful for further investigating population genetics of *A. dahurica*, cultivars and other members of this genus.

## Background

*Angelica dahurica* (Hoffm.) Benth. et Hook. f. ex Franch. & Sav., is an important medicinal plant, whose roots (Angelicae Dahuricae Radix) have been used as the important traditional Chinese medicine “bai zhi” for thousands of years [1]. *A. dahurica* is widely cultivated in North China, who has two cultivars, namely *A. dahurica* cv. ‘Hangbaizhi’ and *A. dahurica* cv. ‘Qibaizhi’. These herbs are thought to have the efficacies of eliminating wind-cold and dampness, expelling pus and relieving pain [2]. In addition to its medicinal uses, *A. dahurica* and its cultivars are also used as raw materials of cosmetics and flavorant [3]. Due to the medicinal and economic values, wild resources of *A. dahurica* were increasingly reduced by over-exploitation and habitat destruction. Meanwhile, the two cultivars of *A. dahurica* have gone through a long time artificial selection, resulting in the decline of the genetic diversity and disease and insect resistance [4, 5]. Gene flow between two cultivars from different producing areas might lead to out-breeding depression, and then, the depressed gene flow goes into the adjacent natural populations of *A. dahurica* which might cause its decline in the adaptability to local environment [6]. Wild resources usually have high genetic variation and are valuable germplasm. One of the keys to biodiversity conservation is to protect species, more specifically, to protect the genetic diversity or evolutionary potentials of species. Therefore, in order to ensure the sustainable utilization of *Angelica dahurica* resources, conservation genetics research should be strengthened to protect the genetic diversity of this species as much as possible. The assessment of genetic diversity and population genetic structure in *A. dahruica* could help to formulate scientific and effective protection strategies.

Microsatellite markers are simple sequences repeat (SSRs) consisting of 1–6 nucleotides in length (motifs) and can be found in genomes of all prokaryotes and eukaryotes [7]. During the past decades, microsatellite markers, due to the high level of polymorphism, high abundance, co-dominance, selective neutrality and transferability across species [8–12], have been widely applied to a variety of biological researches, such as population genetics, gene flow, phylogenetics, and conservation genetics. A latest study has reported 18 polymorphic loci isolated from *Angelica sinensis* [13]. However, no microsatellite loci were successfully applied to *A. dahurica*, which might have resulted from low success rate of amplification or no polymorphism. Therefore, it is necessary to develop novel microsatellite makers to conduct the population genetics on *A. dahurica*.

Next-generation sequencing technology is a powerful, cost-effective, and reliable tool, generating a large number of sequence data, which can assist us to develop SSR markers [14–17]. Especially, RNA-Seq with Illumina sequencing can help researchers in the identification and development of a large number of SSR markers, which is faster, easier, and more cost-effective compared to traditional SSR development processes [18]. This study aims to use the RNA-Seq technology to develop novel microsatellite markers which would be useful in evaluating the genetic diversity and population structure of *A. dahurica*. We also hope to use the novel SSR markers as an effective tool for further study on conservations of *A. dahruica*.

## Results

### Assessment of the quality and quantity of RNA/DNA

The concentration and purity (OD_260/280_) of extracted RNA from the sample (Voucher no. 2017826-CD-X) was 256.7 ng/ul and 2.02, respectively, which were satisfied for the requirement of Illumina sequencing. DNA extracted from 89 individuals of four populations were also qualified, whose concentration and purity (OD_260/280_) ranged from 38.6 ng/ul to 398.3 ng/ul and from 1.64 to 1.99, respectively.

### SSR markers screening and polymorphisms assessment

In total, RNA-Seq yielded 85,000,882 clean paired-end reads at least 150 bp in length, and 85,650 unigenes were gained from the clean reads performed by de novo assembly with Trinity. MISA totally identified 11,289 putative SSRs. According to this detailed information, 9436 (83.6%) primer pairs were designed with the program Primer3 and assessed loci containing di-, tri-, tetra-, penta-, and hexanucleotide repeat motifs. Thirty loci were randomly selected to amplify 5 samples from each of the four populations. However, 14 out of the 30 loci failed to amplify the test samples or performed non-specific amplification. The remaining 16 loci (Table 1) could amplify and yielded polymorphic amplification products in the four populations (Table 2, Additional file: Table S1).

**Table 1:** Characteristics of 16 microsatellite primers developed for *Angelica dahurica*

Locus	Forward and reverse primer sequences	Motifs	product Size range (bp)	***T***_***a***_	GenBank Accession No.
AD1	F:TCCTCCAGCTGGCATAATAATAA R:ATTAAAAAGAACAAGGGGCTCAA	(TGC)6	111–123	55	MH220032
AD2	F:CTTTTTCCCCAATTCCTATCAAC R:CACACCCGTAGAGATACACACTG	(TA)9	136–188	55	MH220033
AD4	F:TTAACAACTGGCTTTGATCCATT R:TGGAGGTTTGTACGATTTTATGG	(TG)9	90–126	50	MH220034
AD7	F:GCTCTCTTAAATTTCACCCCAAC R:TACTAGATTCTTCCAGAGCGACG	(ATTACC)4	131–155	55	MH220035
AD8	F:TTCAACATGGTCATGTGAGTGAT R:CCGTTGGAGGTCTTCTTGTAAAT	(GGAGTG)4	140–164	53	MH220036
AD9	F:CAACACACATGATCCAGAAGAAA R:GAGCTGGAGATAGTCTGTTGCAT	(TCTGCA)10	99–159	50	MH220037
AD10	F:AGACTGCACCTGTCTCATTTTTC R:GGCTTGTAATTAATCTTTGCACC	(GT)9	116–140	50	MH220038
AD11	F:TTCGTCATTTAGAAACGATAGCA R:TCAATGGATACCACCACATCATA	(TCT)7	127–142	50	MH220039
AD12	F:AAGTTCAATATTCCCCTGGTGAC R:GTACATTATACCCTCCCCAAACC	(AAACC)4	126–131	55	MH220040
AD14	F:TGTACTCCATGGACTGGAGTCTT R:TTTGTTTTCTGACAAAGCCAAAT	(TCA)7	108–123	50	MH220041
AD17	F:GGATCATGTTGATGATGGAAAAT R:TTCGATTACTACAGCAGATGAGC	(AGA)7	145–163	50	MH220042
AD19	F:CCCCATTTCTCCCATAGATAGAT R:CCATTAATTGTTCTGCATTTTCC	(GCA)6	125–140	53	MH220043
AD22	F: AAACAATATCAAATCAAATGGCGR: GTGGTGATGATGAATCTTGTGAA	(TTC)6	84–99	50	MH844986
AD23	F: GCTTGACATATATCATCGCCTTTR:TAGACCAAGAGCCAAATAAACCA	(CTT)_6_	130–142	50	MH844987
AD24	F: GCGAGATGGAAATGACAAATTCTR: ATCCCACCATTTCCTCATTAAGT	(GCA)_6_	108–117	50	MH844988
AD26	F: ACAAACAAACCAAATCAATCTCGR: TTATCTTCCAATTGCTTGTTGCT	(CAAAC)4	118–123	50	MH844989

**Table 2:** Genetic diversity parameters obtained with 16 microsatellite loci across four populations of *Angelica dahurica*

		BX pop (N^**a**^ = 24)			AS pop(***N*** = 17)			KS pop(***N*** = 24)			CD pop(N = 24)	
Locus	***N***a^**b**^	***H***o^**c**^	***H***e^**d**^	***Na***	***H***o	***H***e	***N***a	***H***o	***H***e	***N***a	***H***o	***H***e
AD2*	9.00	0.667	0.747	9.00	0.733	0.829	4.00	0.435	0.504	8.00	0.522	0.768
AD10*	7.00	1.000	0.712	7.00	1.000	0.785	4.00	0.917	0.687	7.00	0.870	0.684
AD8	5.00	0.667	0.541	4.00	0.294	0.263	3.00	0.333	0.603	4.00	0.478	0.398
AD7	3.00	0.417	452	3.00	0.412	0.403	2.00	0.250	0.219	1.00	0.000	0.000
AD9*	7.00	0.750	0.743	3.00	0.353	0.552	5.00	0.542	0.685	8.00	0.667	0.600
AD1	4.00	0.417	0.506	4.00	0.294	0.306	3.00	0.417	0.338	2.00	0.167	0.153
AD17*	5.00	0.667	0.739	4.00	0.765	0.657	3.00	0.625	0.633	4.00	0.625	0.563
AD14*	5.00	0.917	0.576	5.00	0.882	0.619	4.00	0.917	0.608	2.00	0.000	0.080
AD4*	6.00	0.917	0.731	4.00	0.765	0.673	5.00	0.208	0.594	3.00	0.042	0.155
AD11	4.00	0.667	0.595	3.00	0.588	0.552	4.00	0.583	0.688	3.00	0.500	0.531
AD12	2.00	0.458	0.499	2.00	0.353	0.457	2.00	0.304	0.258	2.00	0.348	0.287
AD19	5.00	0.583	0.611	4.00	0.706	0.734	3.00	0.583	0.569	4.00	0.625	0.526
AD22	5.00	0.375	0.389	4.00	0.765	0.512	5.00	0.792	0.576	6.00	0.750	0.621
AD23*	1.00	0.000	0.000	2.00	0.400	0.444	2.00	0.087	0.287	3.00	0.042	0.155
AD24*	4.00	0.421	0.580	3.00	0.615	0.521	4.00	0.652	0.509	3.00	0.833	0.531
AD26	2.00	0.304	0.258	2.00	0.059	0.057	2.00	0.458	0.353	1.00	0.000	0.000
Average	4.63	0.577	0.543	3.94	0.562	0.523	3.44	0.506	0.507	3.81	0.404	0.378

^a^*N* = number of individuals sampled; ^b^*N*a = number of alleles; ^c^*H*o = observed heterozygosity; ^d^*H*e = expected heterozygosity. Asterisk (*) indicates the loci significantly departed from HWE (*P* < 0.05)

The number of alleles ranged from 2 to 15 per polymorphic locus over the four populations (Table 2) while observed and expected heterozygosities ranged from 0.000 to 1.000, with average 0.512 and from 0.000 to 0.829, with average 0.488, respectively. Significant deviations from HWE were observed at 8 loci (Table 2). Deviation from HWE likely resulted in null alleles, which were detected in 68.8% of these loci, according to the Micro-Checker analyses. Tests of linkage disequilibrium were significant for 11 pairs of loci across all populations (AD9 and AD10, AD9 and AD8, AD9 and AD17, AD9 and AD11, AD11 and AD19, AD2 and AD4, AD7 and AD14, AD8 and AD11, AD10 and AD11, AD1 and AD11, AD23 and AD24) (*P* < 0.01). After sequential Bonferroni correction, none of the informative locus pairs at BX、AS、CD population was significant while at KS population, five pairs were significant (AD8/AD9; AD10/AD9; AD11/AD9; AD17/AD9; AD11/AD19). These results indicate that the newly developed microsatellite primers will be useful for further investigating population genetics of *A. dahurica* and other members of this genus.

### Cross-species amplification

The 16 polymorphic SSR loci isolated from *A. dahurica* were tested for amplification in 10 individuals of *Angelica dahurica* cv. ‘Hangbaizhi’ and 5 individuals of *Angelica decursiva*. The results showed that 14 of the 16 SSR loci (87.5%) had transferability in both *A. dahurica* cv. ‘Hangbaizhi’ and *A. decursiva*, respectively (Table 3).

**Table 3:** Cross-amplification results for the 16 SSR loci developed from *Angelica dahurica* in *Angelica dahurica* cv ‘Hangbaizi’ and congeneric *Angelica decursiva*

		Size ranges	
Locus	*Angelica dahurica* ‘Hangbaizhi’ (NZ pop)	*Angelica dahurica* ‘Hangbaizhi’ (XY pop)	*Angelica decursiva* (QY pop)
AD1	114–120	114–120	117
AD2	/^a^	/	/
AD4	100–102	96–102	80–104
**AD7**	131–137	131–143	201
AD8	146	146–152	140
AD9	99–117	99–117	99
AD10	116–138	116–132	102–114
AD11	127	127–136	133
AD12	106	/	91–121
AD14	102–120	117–120	114
AD17	154–157	154–160	142
AD19	125	125–128	128–134
AD22	87	87	90–93
AD23	142	142	133
AD24	111–114	111–126	108–111
AD26	/	/	/

^a^(“/”) indicates failure in cross-species amplification

## Discussion

### Methods for development of SSR markers in plant

During the past decades, many methods of developing SSR markers have arisen, such as, (1) screening SSRs from the genomic DNA library, (2) enriching by magnetic beads, (3) PCR-based isolation of SSR arrays, (4) searching SSRs in GenBank, EBML or DDBJ [18, 19]. The methods were labor-intensive, cost and time-consuming, as some had to construct genomic DNA library and screen the libraries many times for different SSR sequences and the yield of positive clones was very low; or some had to know the genomic information for designing primers; or had to use sophisticated enzyme restriction technique, or lack information on targeted species in database [20, 21].

With the rapid development of sequencing technology during the past decade, next-generation sequencing (NGS) technology has made RNA-seq more effective and reliable [18]. An amount of sequence data can be produced by RNA-seq which will be used to develop novel molecular markers [16]. RNA-Seq is time and labor saving, and so effective and economical that can almost overcome the drawbacks of traditional methods cited above. On the other hand, this method is especially applied for species without a reference genome in molecular markers development [22]. Additionally, high stability among technical replicates makes RNA-Seq data more useful [23]. RNA-seq presently has been applied for EST-SSR development in many plant species, such as *Rosa roxburghii* [24], *Neolitsea sericea* [25], *Salix* [26], *Elymus sibiricus* [27]*, Xanthoceras sorbifolia* [28], etc. In this study, a total of 11,289 putative SSRs were identified. These large dataset resources would be useful in studies on the genetic diversity, and population genetics of *A. dahurica*. We also hope to use the novel EST-SSR markers as an effective tool for further studies on conservations of *A. dahruica*. Firstly, with the EST-SSR markers, we can evaluate the genetic diversity of *A. dahurica* and cultivars in a large scale and protect their germplasm resources as much as possible. Secondly, combining the genetic information produced by SSRs and ecological data may help finding out the impact factors on the increasing decline of this wild resources and implement effective protection measures of this species and its genetic diversity. We can also evaluate the population genetic structure for determining core collection areas as the protection sites or ranges in future, etc.

### Transferability of cross-species amplification

SSR markers have shown transferability in different species, especially for the closely related species. The degree of transferability relays on the conserved degree of flanked sequences and evolutionary stability of SSR [29]. Xu and Li used 66 polymorphis loci of *Liriodendron tulipifera* in the amplification of *Liriodendron chinense* and *Michelia alba* [30]. They found transferability rates of 85 and 54%, respectively. Han et al. randomly screened 10 SSRs for the genus of *Salix* and *Populus*. The amplification results suggested 14 SSRs were universal in both genus [31]. Zheng et al. firstly demonstrated the genomic SSRs of Dicotyledons (Genus *Gossypium*) could be used in Monocotyledons (*Musa nana*), and they also found the transferability of EST-SSR was much stronger than genomic SSR [32]. All of these studies suggested not only that SSR markers have shown transferability in closely related species, but also in different genus, even different family. Before our study, although the development of a congeneric species (*Angelica sinensis*) SSRs had been published [13], the authors found these markers failed to amplify or had no polymorphism in *Angelica dahurica* by pre-experiment. The newly developed EST-SSRs here showed 14 out of 16 polymorphic loci in *A. dahurica* had transferability in cultivars and closely related species (*A. decursiva*). Based on the transferability, the gained SSR markers would improve the efficiency and reduce the cost in marker development, and increase the number of SSRs in genus *Angelica.* Especially for the species of genus *Angelica* with little genomic background information, making use of the SSRs of closely related species for developing SSR markers would be very convenient. In this study, according to the cross-species amplification test, we also found the product size range (131-143 bp) of locus AD7 in *A. dahurica* (including *A. dahurica* cv.”Habaizhi”) differentiated with that (201 bp) of *A. decursiva*. Locus AD7 would be a potential diagnostic marker for molecular identification of *A. dahurica* and *A. decursiva*.

## Conclusions

The newly developed 16 EST-SSRs of *A. dahurica*, achieved by RNA-Seq data analysis in this study would be potentially useful in further investigating the population genetics and genetic diversity of *A. dahurica,* its cultivars and congeneric species. The diagnostic marker (Locus AD7) would be useful in the application of identification of *A. dahurica* and *A. decursiva*.

## Methods

### Plant materials

We sampled *Angelica dahurica* (Hoffm.) Benth. et Hook. f. ex Franch. & Sav. from 4 populations from its natural distribution area throughout the central (Chengde, He bei province) and northeastern part (An shan and Benxi, Liao Ning province; Liuhe, Ji Lin province) of China (Details of sampling information and voucher numbers of specimens were shown in Table 4). Within populations, sampled plants were separated by at least 20 m to avoid multiple samples from the same clone. In each population, fresh leaves from 17 to 24 individuals were collected and preserved in gel–dried silica until DNA extraction. Totally, 89 individuals were selected to assess polymorphisms of the developed microsatellite markers. Ten specimens of *A. dahurica* cv.’Hangbaizhi’ and 5 specimens of *Angelica decursiva* (Miq.) Franch. et Sav. were used for cross-species amplification (Table 4). A specimen (Voucher no. 2017826-CD-X) was collected from CD population (Chengde, Hebei province of China) and then immediately frozen in liquid nitrogen for RNA extraction. Permissions were not necessary for collecting these samples, as they did not distribute in nature reserves and also this species has not been included in the list of national key protected plants. All specimens above were morphologically identified by associate professor Enwei Tian from School of Traditional Chinese Medicine, Southern Medical University (SMU). The voucher specimens were deposited in the herbarium of SMU (Table 4). Our field study and Experimental research complied with local legislation, national and international guidelines. The authors also complied with the Convention on the Trade in Endangered Species of Wild Fauna and Flora.

**Table 4:** Locality and voucher information for the populations of *Angelica dahurica*, *Angelica dahurica* cv. ‘Hangbaizhi’ and *Angelica decursiva*

Species	Voucher no.	Collection locality	Geographic coordinates	Sample sizes
*Angelica dahurica*	2,017,811-BX(1 ~ 24)	Benxi (BX)	44°22′ 47″ N and 124° 57′ 58″ E	24
	2,017,808-AS(1 ~ 17)	Anshan (AS)	41°00′ 55″ N and 123° 08′ 05″ E	17
	2,017,812-KS(1 ~ 24)	Tonghua (KS)	42°25′ 49″ N and 126° 06′ 36″ E	24
	2,017,826-CD(1 ~ 24)	Chengde (CD)	40°40′ 12″ N and 117° 40′ 12″ E	24
*Angelica dahurica* cv.’Hangbaizhi’	2,015,830-NZ(1 ~ 5)	Nanjing (NZ)	32°03′ 41″ N and 118° 50′ 27″ E	5
	2,016,813-XY(1 ~ 5)	Xianyang (XY)	34°20′ 05″ N and 108° 42′ 25″ E	5
*Angelica decursiva*	2,017,925-QY(1 ~ 5)	Qiangyuan (QY)	24°33′ 33″ N and 112° 05′ 37″ E	5

### RNA/DNA extraction, cDNA library construction and Illumina sequencing

Specimen (Voucher no. 2017826-CD-X) collected from CD population was used to extract total RNA with a modified CTAB method [33]. For detecting the polymorphisms of isolated microsatellite loci, eighty nine specimens were used for genotyping (Table 4). Before that, genomic DNA of these specimens was extracted using a modified CTAB method [34]. The quality and quantity of the exacted RNA/DNA were assessed using a NanoDrop 1000 UV/Vis spectrophotometer (Thermo Scientific, Wilmington, DE, USA) and 1.5% agarose gel electrophoresis. Illumina TruSeq RNA Sample Preparation Kit (Illumina, San Diego, California, USA) was used for constructing RNA-Seq library. Construction of cDNA library and Illumina sequencing on HiSeq 4000 platform were conducted by TGS (Shenzhen, China).

### SSR markers development, PCR amplification and polymorphic loci screening

The raw data yielded from RNA-Seq was firstly conducted with filtering and quality control using Qiagen CLC Assembly Cell v.4.2.1, and then performed de novo assembly with Trinity v2.4.0 [35, 36]. In order to mine and identify SSR loci, the MicroSAtellite tool (MISA,v1.0) was conducted to hunt putative microsatellite motifs in gained unigenes [37]. The unigenes were searched for 1–6 nucleotide repeat motifs. Mononucleotide repeats were set to extend at least 10 repeats, dinucleotide repeats extend at least six repeats, and repeats of all other motif lengths extend at least three repeats [38]. From the unigenes, SSR primers were designed using Primer 3 according to the criteria given by Tian et al.: (1) product sizes of the PCR amplification between 80 and 300 bp; (2) primer length of 18–25 nucleotides; GC content of 40–55%; (3) annealing temperature 5 °C lower than the Tm value (55–65 °C) [39, 40]. After primer designing, we randomly selected 30 pairs of primers under the condition of having targeted product sizes between 80 and 250 bp. Di-, tri-, tetra-, penta-, and hexanucleotide repeat loci have at least 9, 6, 5, 4, 3 repeats, respectively. The primers were synthesized in Invitrogen company (Shanghai, China).

Subsequently, these 30 primer pairs were tested for proper PCR amplification in *A. dahurica* using total genomic DNA (Five samples from each population were tested for PCR). The amplification reactions were carried out with 2720 thermal cycler (Applied Biosystems, Foster City, CA) in 20 μl volume containing 20 ng of genomic DNA, 0.2 mM of each dNTP, 0.4 μM of each primer, 10 × PCR buffer (Mg^2+^ free), 2.5 mM Mg^2+^, 1 unit of Taq DNA polymerase (Takara, Dalian, China) with the following conditions: initial denaturation at 95 °C for 5 min, followed by 35 cycles of 94 °C, 30 s; 50 to 55 °C, 60 s; 72 °C, 45 s and a final extension of 72 °C for 8 min. The PCR products were visualized at 1.2% agarose gel electrophoresis. Primers that successfully amplified were then selected for assessing polymorphisms. Before that, the primer pairs were labeled with fluorescent dyes (TAMRA or FAM) at forward primers (5′ end). To characterize the genetic polymorphism of these microsatellite markers, genomic DNA of the 89 *A. dahurica* individuals from the four natural populations were used. PCR reactions and cycling conditions were performed as above. The fragment sizes of the PCR products were determined on the ABI PRISM 3100 Genetic Analyser (Applied Biosystems, Foster City, CA) using Genotyper 4.0 and LIZ 500 (Applied Biosystems, Foster City, CA) as an internal size standard.

### SSR markers data analysis

Possible null alleles, large allelic dropout and genotyping errors in the microsatellite loci were detected with Micro-Checker version 2.2.3 [41]. The diversity indexes, which include the number of alleles (*Na*), observed (*Ho*) and expected heterozygosities (*He*) per locus and population were estimated using GENALEX version 6.1 [42]. We conducted the test for departures from Hardy–Weinberg equilibrium of the SSR loci using the Markov chain method (settings: dememorization: 1000; batches: 100; iterations per batch: 1000) in Genepop 4.0 [43]. Tests of linkage disequilibrium for every pair of loci across all *A. dahurica* populations were also conducted using GENEPOP 4.0. *P*-values were adjusted using the Bonferroni correction [44].

### Cross-species amplification

To validate the transferability of the developed SSRs, the polymorphic loci isolated from *A. dahurica* were tested for amplification in 10 individuals of *Angelica dahurica* cv. ‘Hangbaizhi’ and 5 individuals of *Angelica decursiva* using the same procedures as above, except that the annealing temperature was re-optimized for each locus.

## Supplementary information

**Additional file 1 Table S1**. Raw Genotypic data for all individuals of 16 loci.

## Data Availability

Raw sequence information are available in National Center for Biotechnology Information (NCBI) Sequence Read Archive (RNA sequence information, SRA: SRP162120 BioProject ID PRJNA490770). Sequence information for the developed EST-SSR primer pairs has been deposited at GenBank (Accession numbers are provided in Table 1). Other datasets supporting the conclusions of this article are included within the article and its additional file.

## Abbreviations

EST-SSR: Expressed sequence tag-Simple sequence repeat marker
RNA-Seq: RNA sequencing
MISA: MicroSAtellite
HWE: Hardy-Weinberg equilibrium
NGS: Next-generation sequencing
CTAB: Cetyltriethylammnonium bromide
*Na*: Number of alleles
*Ho*: Observed heterozygosities
*He*: Expected heterozygosities
